# Structural Features of Triethylammonium Acetate through Molecular Dynamics

**DOI:** 10.3390/molecules25061432

**Published:** 2020-03-21

**Authors:** Enrico Bodo

**Affiliations:** Chemistry Department, University of Rome “La Sapienza”, Piazzale Aldo Moro 5, 00185, Rome Italy; enrico.bodo@uniroma1.it

**Keywords:** molecular dynamics, semi-empirical methods, ionic liquids

## Abstract

I have explored the structural features and the dynamics of triethylammonium acetate by means of semi-empirical (density functional tight binding, DFTB) molecular dynamics. I find that the results from the present simulations agree with recent experimental determinations with only few minor differences in the structural interpretation. A mixture of triethylamine and acetic acid does not form an ionic liquid, but gives rise to a very complex system where ionization is only a partial process affecting only few molecules (1 over 4 experimentally). I have also found that the few ionic couples are stable and remain mainly embedded inside the AcOH neutral moiety.

## 1. Introduction

Protic ionic liquids (PILs) [1,2,3] are members of the class of materials known as ionic liquids (ILs), which are highly ionized substances with melting temperatures below 100 °C. PILs can be formally thought of (and often practically synthesized) as the result of an acid–base reaction, where the ionization occurs because of proton transfer. The cohesive energy in these liquids stems from both the Coulomb interactions and hydrogen bonding. Typically, only a large difference in the pK_a_ of the reagents (>6) guarantees that the ensuing liquid is completely ionized [4]. If the difference in pK_a_ is small (<4) [5], the liquid may turn out to be a mixture of ionic and polar moieties, where phase separation and evaporation of the volatile component may occur to various extents—a situation that is not auspicial for practical applications, but underlines an intriguing chemical complexity.

Despite their apparent simplicity [6], PILs can sometimes show complex structural patterns and unexpected chemical activity owing to a series of inter-related phenomena such as nano-segregation [7,8], hydrogen bonding [9,10,11,12,13], and tautomerization reactions involving secondary protic functions on the molecular structure [14,15]. The latter, in turn, may spawn proton mobility [16,17]. A recent work [18] on “quasi-ionic” materials containing acetic acid provided evidence that an incomplete ionization in certain PILs can enhance their conductivity. Additionally, recent studies on various ILs and PILs [19,20,21,22,23,24] have demonstrated the existence of like-charge molecular aggregates, an issue that complicates their structure even further.

The mixture of triethylamine (TEA) and acetic acid (AcOH), formally triethylammonium acetate [TEAH^+^][AcO^−^], is a commercially available liquid that is routinely used as solvent in organic synthesis. As it comes from the combination of Bronsted acid and base, it is often referred to as a protic ionic liquid. However, the difference in pK_a_ of the two components is only 5 units, thereby letting us surmise that its real physical state might be a mixture of an ionized and a neutral phase. It is thus instructive to study such a relatively simple system in order to understand the behavior of those substances that lie at the border of the ionic and polar regimes. Two papers [25,26] have appeared recently in which a set of experimental determinations of this liquid have been performed. The former presented an analysis by means of IR spectral measurements; the latter investigated in more detail the structure of the liquid. In both papers, it seems clear that the mixing of acetic acid with triethylamine does not produce a completely ionized medium. Part of the polar component (triethylamine) separates and leaves a homogenous liquid that is composed by a portion of ionic liquid dissolved in acetic acid. The composition of the liquid, as stated in [26], seems to be given by the rough formula [AcO^−^][TEAH^+^][AcOH]_3_, where the excess TEA has separated.

## 2. Computational Methods

It is well known [27] that PILs, owing to hydrogen bonding and polarization effects, are hard to model correctly using force fields based on fixed atomic charges. In order to perform molecular dynamics (MD), either a polarizable force field has to be employed, or electron density has to be explicitly calculated to account for fluctuating charges and many-body effects. It has been shown, ab-initio or semi-empirical MD [28] are able to properly describe hydrogen bonding features at very good levels of accuracy. Owing to the size of the simulated systems and to the timescales involved in the proton transfer process, I have chosen a semi-empirical approach and, in particular, the density functional tight binding (DFTB) method. There are several advantages in using semi-empirical MD over ab-initio computational schemes or classical MD. First, the performance of the DFTB method is at least one order of magnitude faster than an ab-initio. Second, unlike classical MD, the chemical topology of the molecules is not enforced during the simulations, thereby allowing for bond breaking and proton transfer. Finally, polarization effects are naturally accounted for by the presence of the fluctuating atomic charges.

The DFTB+ [29] package with the *3ob* [30] parameter set has been used for molecular dynamics simulations of the bulk phase. Third-order corrections (SCC-DFTB3) [31], with self-consistent charges, have been used during the production trajectory along with the D3 dispersion corrections. Owing to the relatively long timescales necessary to equilibrate the systems (because of the slow proton transfer processes to occur), I have performed five MD simulations using different cell sizes and initial conditions:

(1) **20:20**, a simulation where a cubic cell with side length of 17.2 Å was initially made by 20 AcOH/TEA neutral pairs. NVT MD was run at a constant temperature of 300 K for a production time of 300 ps. The density was set to 1.04 g/cm^3^;
(2) **10:10**, a simulation where a cubic cell with 13.6 Å side length contained 10 AcOH/TEA initially neutral pairs. NVT MD was performed at 300 K for 600 ps. The density was set to 1.04 g/cm^3^;
(3) **8:2**, a simulation prepared with a 4:1 AcOH:TEA composition (to account for TEA separation/evaporation detected by experiments [27]) with two TEA molecules and eight AcOH in their neutral state. The cell side was set to 10.3 Å. Production time on this system was 950 ps at 300 K. The density was set to 1.02 g/cm^3^;
(4) **PI20:20,** a simulation with a *partially ionized* composition [AcOH]_3_[TEA]_3_[AcOH]^−^[TEAH]^+^, where one fourth of the molecules are initially ionized. The cell size was set to 17.3 Å and the MD was performed for 300 ps of production time at 300 K. The density was set to 1.03 g/cm^3^;
(5) **I20:20**, a simulation with a completely *ionized* equimolar composition [AcOH]^−^[TEAH]^+^, whose size is identical to the **20:20** one, but all TEA molecules are initially protonated and all AcOH are deprotonated. Production was 300 ps. This simulation was run at both 300 K and 360 K.

In all five simulations, the initial packed configurations were obtained by running a classical MD with the MM3 force field in the NPT ensemble. For all the simulations, the DFTB equilibration time equals one-third of the stated production one.

The limits of the current approach lie in the semi-empirical nature of the Hamiltonian and in the relatively short simulation time, which does not ensure the reaching of an equilibrium condition for the proton migration processes (this would probably take few ns). Using different initial conditions and by looking at their non-equilibrated evolution, I was able to clearly identify the fate of the system.

A minimum energy path connecting the neutral and ionic state of the AcOH/TEA pair was calculated by means of DFT using the Orca code [32]. I started placing the proton on the O atom of the AcOH molecule and gradually moved it toward the N atom of the TEA one. The starting geometry was found by optimizing the neutral state of the pair because, in vacuo, the ionized state is unstable. Two different geometric scans were computed: in the first one, the O–H distance of the AcOH molecule was varied from 1.0 to 1.7 Å in 15 steps and the rest of the system was left free; in the second one, the same procedure was applied, but another constraint was activated to keep the O–N distance fixed at its equilibrium value of 2.65 Å. The geometric scans were computed using a constrained optimization with the M062X functional (chosen because of its suitability in describing charge separated species owing to the high percentage of exact exchange) and the def2-SVP basis set. For each scan, a total of 15 geometries was obtained and, for each of them, single point energy calculations with both M062X/def2-TZVP and DFTB were carried out. Dispersion corrections [33] were included in both the ab-initio and DFTB calculations.

## 3. Results and Discussion

### 3.1. Validation with Available Data

The DFTB method has been successfully applied to a large number of chemical systems ranging from metallic nanoparticles to ionic liquids [34,35,36]. The method has been proven to reproduce both structural and energetical properties of protic ionic liquids.

In order to validate the method as far as possible, I computed the proton affinities (PAs) of the relevant molecules with the SCC-DFTB3 and compared them to experimental values. A correct evaluation of the PA is indeed a crucial test of the ability of the employed semiempirical scheme to reproduce the thermodynamic of the proton detachment/attachment processes that I expect to be the leitmotiv in the simulations. The PA of acetic acid and TEA is computed as the following energy differences:

PA = E(AcO^–^) + E(H^+^) − E(AcOH)  and  PA = E(TEA) + E(H^+^) − E(TEAH^+^)

In the DFTB framework, one has to account for the fact that the electronic energy of the proton is not zero. For the present computations and according to the DFTB3 paradigm, I adopted the value of 151.04 kcal/mol for the proton energy [34] The computed PAs for AcO^–^ and TEA are 356 and 240 kcal/mol, respectively, which compare favourably well with the literature values of 355 kcal/mol for the former [34] and 233 kcal/mol for the latter [37].

**Figure 1:**
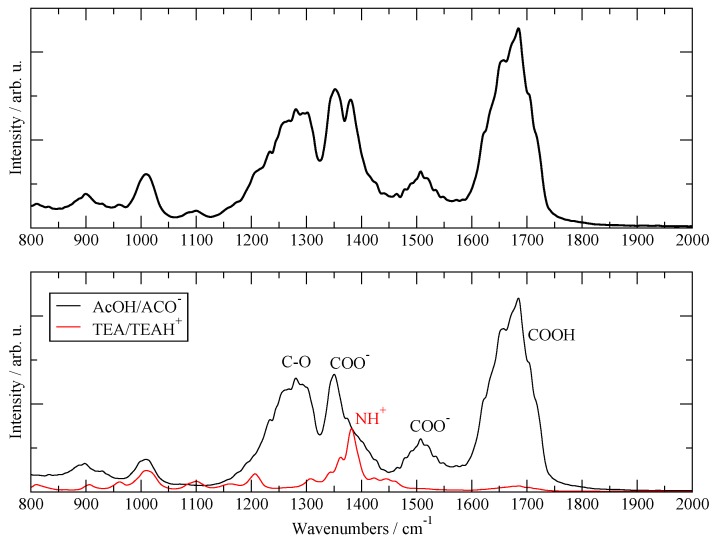
Calculated IR absorption spectra. Top panel: total IR spectra. Bottom panel: contribution from AcOH/AcO^–^ (black) and from TEA/TEAH^+^ (red).

Another validation test of the present simulations was obtained by computing the IR absorption spectra of the liquid using the **20:20** simulation data. The time-dependent molecular and global dipole moments were computed using the fluctuating atomic charges provided by the SCC–DFTB3 scheme and the IR absorption spectrum was generated by a Fourier transform of their autocorrelation functions [38,39,40]. The resulting global simulated absorption spectrum is reported in Figure 1, top panel. The separated contributions due to the two species are reported in the bottom panel, where one can see that the TEA/TEAH^+^ contributes only in a minor way to the IR spectrum (red line). Following the assignment of [25], one can see that the only visible band from TEAH^+^ is the one located between 1350 and 1400 cm^−1^, which is attributed to NH_3_^+^ deformations. The AcOH/AcO^–^ moiety represents the major source of IR absorption in the fluid and contributes to all the major bands in the 1000–2000 cm^−1^ region: one can clearly recognize the C=O absorption due to carboxyl stretching between 1600 and 1700; the C—O stretching of the carboxyl between 1200 and 1300; and the two stretching modes of the carboxylate at 1350 and 1500, which are red-shifted by the presence of H-bonds. The calculated spectrum can be compared with the experimental one reported in [25] in Figure 3. Apart from the intensity of certain bands, the agreement in the position and number of visible bands is very good over the whole fingerprint region.

The low degree of ionization in AcOH/TEA is very likely owing to a combination of enthalpic factors and many body effects, so that the only way to describe the system properly is to perform MD. Anyway, a crucial factor determining the state of the system is the energy difference between neutral AcOH/TEA and ionized AcO^–^/TEAH^+^ when embedded in the bulk. Although this “local” energy difference is not the only driving force in producing or preventing ionization, it is also true that, for the calculations to be reliable, the DFTB method has to reproduce the above enthalpic difference.

**Figure 2:**
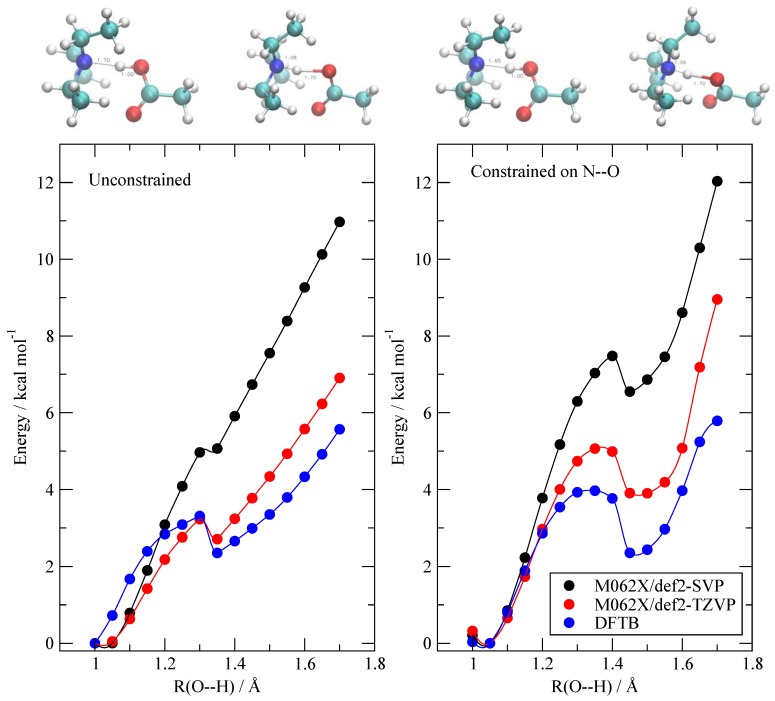
Energy profile of the AcOH/TEA pair as a function of the AcO–H distance (see text for details). The black dots are the M062X/def2-SVP optimized energies. The red ones are M062X/def2-TZVP single point energies, and the blue ones are the density functional tight binding (DFTB) results. Left: scan without any further constraint except the O–H distance. Right: scan with an additional constraint that keeps the N–O distance fixed at its equilibrium value. To allow for comparison, the minimum energy in each set was set equal to zero. The structures on top correspond to the first and last geometries in each scan.

In order to provide such an evaluation, I performed a set of ab-initio calculations on an isolated pair of AcOH and TEA. I built a set of minimum energy geometries connecting the neutral state to the ionized one by gradually moving the proton from the O atom to the N one. The idea is to test if DFTB is able to reproduce the energy variation (as computed by a more accurate method) along the geometric path followed by the pair during the proton exchange. The energy profiles along two possible proton transfer paths were evaluated using both M062X and DFTB, and are reported in Figure 2. The difference in geometries between the two paths (unconstrained and constrained, see Section 2) is only marginal and the results are similar in behavior. As we are in vacuo, the ionized structure AcO^–^/TEAH^+^ (R=1.7 Å) is unstable with respect to the neutral one AcOH/TEA (R = 1.0 Å). The barriers that appear along both paths are merely a consequence of the presence of constraints and are essentially due to the hindered rotation of the AcOH molecule at a fixed O–H distance. The most accurate energies were computed at the M062X level with a TZ quality basis set and are reported in red. The DFTB results are reported in blue. Although the DFTB method slightly underestimates the energy difference between the ionized state and the neutral one, its energies essentially reproduce the M062X ones with an average error of 1 kcal/mol. The DFTB data appear, at least in this case, to be more accurate that the full DFT computation with a smaller (double zeta) basis set (black dots).

### 3.2. The Structure of the Fluid

The results presented here come almost entirely from the **20:20** simulation, and I shall discuss the other simulations separately below. In this simulation, the fluid is characterized by an unexpected complex structure. The first evidence of such complexity is the different aggregation of the two components of the fluid: AcOH and TEA. One can easily spot this difference by computing the center-of-mass radial distribution functions (g(r)) between these components regardless of their charge state.

**Figure 3:**
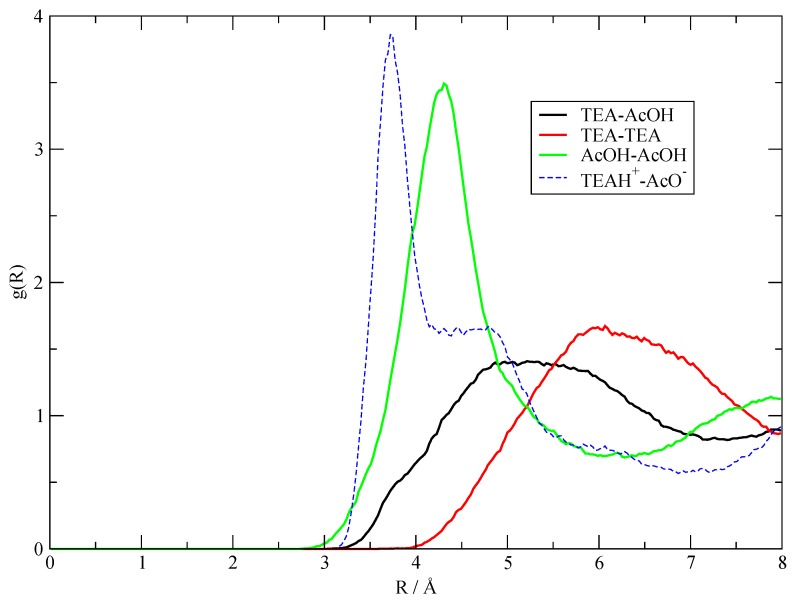
Solid lines: center-of-mass radial distribution functions between the components of the fluid regardless of their charge status as obtained from the **20:20** simulation. The dashed line was obtained from the **I20:20** system to show where the pair distribution would peak in a highly ionized system.

These g(r) are plotted in Figure 3, where I show the radial distribution of AcOH with itself in green; the one for TEA with itself in red; and the mixed distribution, that is, the average distance between the AcOH molecules and the TEA ones, in black. The maximum peak corresponding to the average TEA–TEA distances is obviously placed at larger distances with respect to the other ones because of the larger size of the TEA molecules. Despite this obvious fact, the shape and height of the AcOH–AcOH distribution appear to be radically different from the other two, and this is ultimately because of the strong hydrogen bonding network that AcOH can do with itself. In particular, it is noteworthy to point out that, in the simulations, AcOH tends to cluster more with itself than with TEA molecules, as one can grasp from Figure 3 by comparing the shape of the black and green lines. The g(r) reported in Figure 3 already tell us that the amount of charge transfer in the simulated liquid must be small, because, otherwise, a quantitative proton transfer would give rise to strongly bonded ionic pairs [AcO^–^][TEAH^+^] and increase the short-range values of the black curve in Figure 3 for distances below 4 Å, where a slight shoulder can be noticed. The fact that this is precisely the position of the pair distribution peak in a ionized system is confirmed by the set of data reported as a dashed line, which represents the g(r) between the anion/cation center-of-mass in the **I20:20** fully ionized system (see Section 3.5).

**Figure 4:**
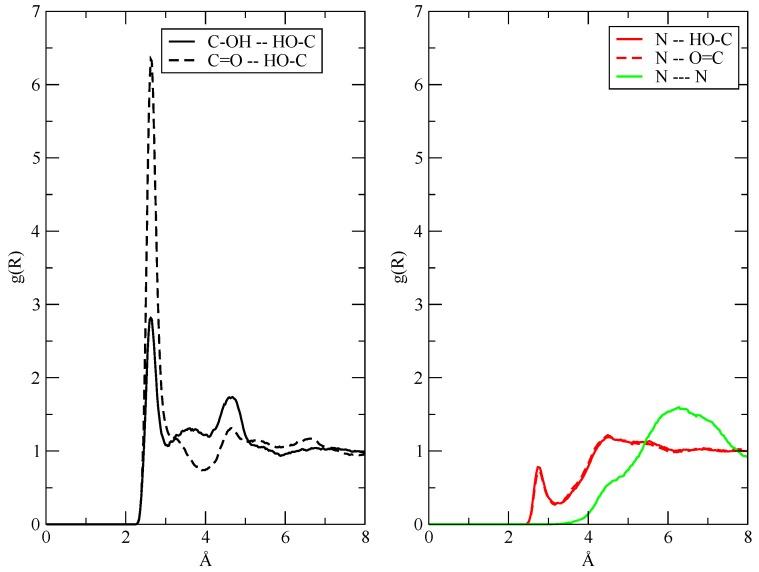
Interatomic g(r) for the H-bonds in AcOH (left, black lines) and for AcOH–TEA interactions (right, red lines). In green, on the right, I have reported the N–N interactions between TEA molecules.

The fact that the H-bonds in the fluids are mainly established between AcOH molecules can also be seen if one looks at the interatomic g(r) reported in Figure 4. In the left panel, I present the acceptor–donor (O—H···O) distance distribution in AcOH. As expected, the H-bond is mainly established between the carboxyl (C=O) and the hydroxyl (—OH) groups. The possibility of the AcOH molecule to act as H-bond donors toward TEA is explored in the right panel, where one sees that the occurrence of O—H···N bonds (red lines) is very limited. The interaction (aggregation) between TEA molecules, whether ionized or not, is substantially a very minor occurrence in the simulations, as shown by the featureless green line in the right panel.

Given these simple results, it is already possible to explain the low ionization degree of the fluid detected by experiments by considering the tendency of the AcOH molecules to cluster together by means of a strong H-bonding network. Evidently, despite the sizable pK_a_ difference between TEA and AcOH, the ionization is partially suppressed by the large cohesive energy within AcOH.

### 3.3. The Dynamics of the Fluid

In this section, I present the dynamics of proton transfer using the **20:20** simulation, and I shall analyze the other datasets further below. Although ionization seems to occur only to a limited extent, it does actually take place in the simulation at least for one AcOH molecule over five (which is not far from the 1:4 ratio of the experimental evidence). I point out that, owing to the rather limited timespan of the simulations, I cannot provide a description of the thermodynamically equilibrated fluid, but I can, nevertheless, describe the outcomes of the proton transfer process.

In the **20:20** simulation, two different proton transfer mechanisms take place: the first occurs within the AcOH moiety, and the second between the AcOH and TEA. In particular, in about 300 ps, I was able to characterize four AcOH→TEA proton transfers. Those taking place within AcOH are more numerous, and I have identified at least seven proton migrations. The two proton migrations mechanisms are very different in nature and provide very different outcomes.

**Figure 5:**
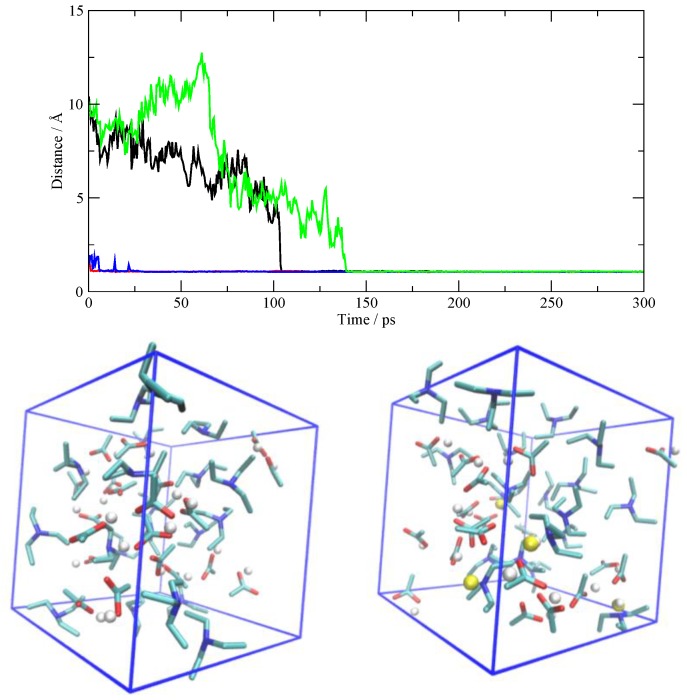
Top: N–H distances as a function of time for the four TEA molecules that acquire the proton from AcOH. Bottom left: the initial simulation cell where the protons attached to AcO are shown as white spheres. Bottom right: the simulation cell at the end of the production (after 300 ps) where four protons have migrated onto TEA (yellow spheres).

**Figure 6:**
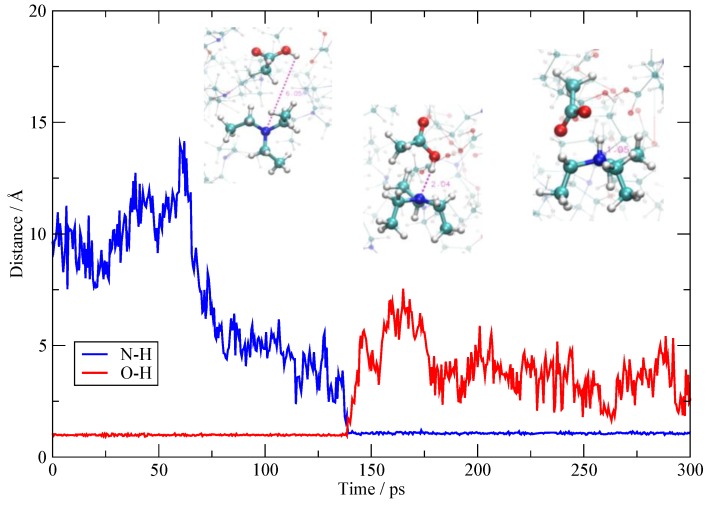
One of the AcOH→TEA proton transfers. The plot reports the O–H and N–H distances (red and blue, respectively). The superimposed snapshots show the geometric configurations of the involved molecules along the dynamics (around 75, 130, and 200 ps).

The former situation (AcOH→TEA) is exemplified in Figure 5, where I have reported the N–H distances as a function of time. Only the distances pertaining to the four protonated TEA molecules are shown. Two of the TEA molecules are protonated right at the beginning of the production phase (red and blue curves), while it takes between 100 and 150 ps to protonate the other two. In the lower panel, I have drawn the initial and final situation of the simulation cell and I have highlighted in yellow the transferred protons, which are otherwise drawn in white. A more detailed view of the AcOH→TEA proton transfer is described for one occurrence in Figure 6. Initially, the proton is on the AcOH at a distance of about 10 Å from the nitrogen atom. At 130 ps, one sees the proton migration. After the proton transfer, the ensuing carboxylate tends to remain bound to the ammonium group by means of an H-bond. In all the cases detected in the simulation, the proton transfer is irreversible, and the two molecular ions, after their creation, are stable along the remaining span of the trajectory.

**Figure 7:**
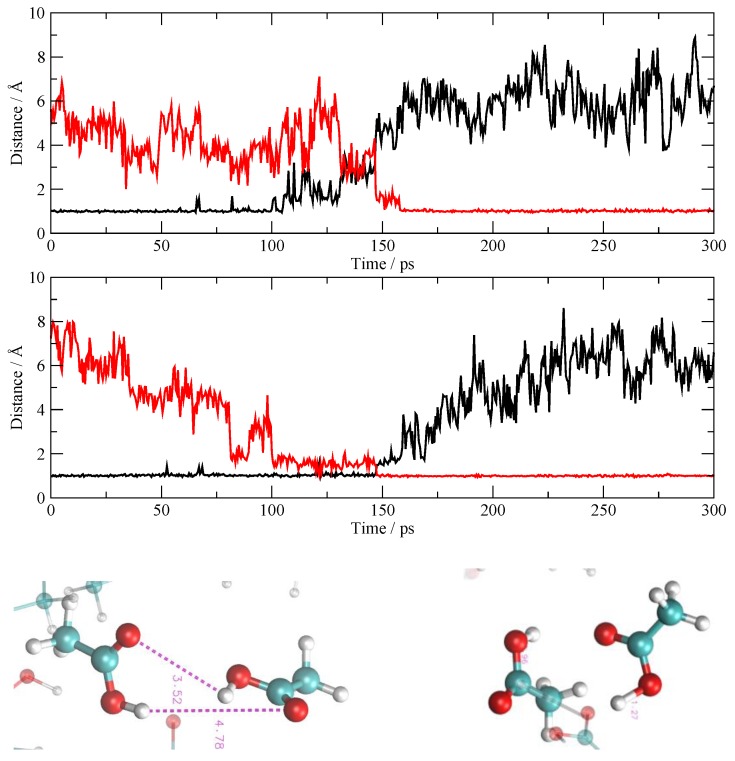
One of the double AcOH→AcOH proton transfers. The two plots report the four O–H distances involved, in red and black for acceptors and donors, respectively. The two snapshots below show the geometric configurations of the involved molecules before and after the transfer.

Proton migration between AcOH molecules is quite well known and takes place within the AcOH aggregates that are being formed in the system. Different from the previous situation, the AcOH portion of the fluid tends to remain neutral and to maintain a low degree of ionization. The net result is that one proton exchange is always accompanied by another one that takes place almost simultaneously in order to maintain the neutrality of the partaking molecules. In Figure 7, I report one example of two O–H pairs of distances (donors and acceptors) for two molecules involved in the exchange. As I have said above, the two proton migrations take place simultaneously around 150 ps, hence they do not lead to ionization, but the loss of a proton is balanced by the acquisition of another one.

This short description of the dynamical processes inside the fluid agrees qualitatively with the experimental picture described in [26]. A large portion of the liquid tends to remain neutral and only a fraction of it, corresponding to a molar ratio of approximately 1:5, is actually made by ionized [TEAH^+^][AcO^–^] pairs. The liquid turns out to be a mixture of a neutral phase that corresponds to an aggregated fluid of AcOH and two dispersed phases—one is made by ionic pairs and the other by neutral TEA molecules. In the simulations, proton migrations take place all over the fluid, but those within the AcOH moiety do not lead to any net charge transfer and leave the AcOH in its neutral state. Only few AcOH molecules transfer the proton to a corresponding TEA molecule. These proton migrations (as far as I have seen within the time span of the simulations) are stable.

### 3.4. The Fate of the Ionic Pairs

**Figure 8:**
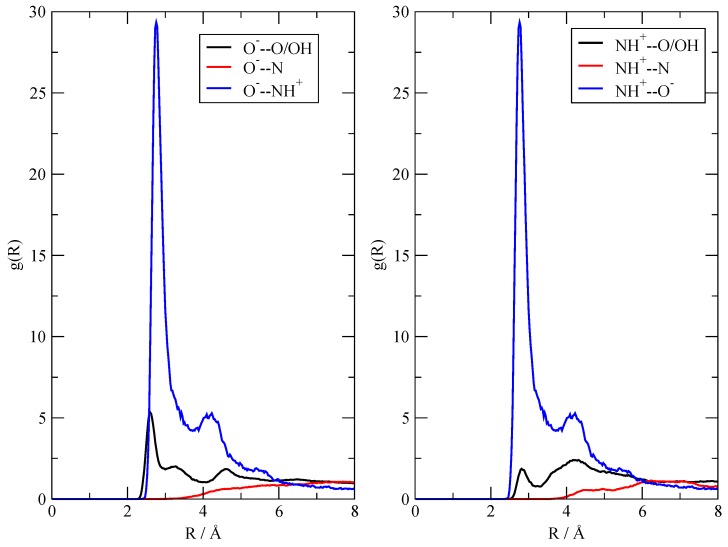
Radial distribution functions characterizing the immediate surroundings of two of the [TEAH^+^][AcO^–^] ionic pairs in the **20:20** simulation.

Although the calculations already qualitatively agree with the experimental observations (in that they show ionization only to a limited extent), I was able to further characterize the fluid by studying the immediate surroundings of the few ionic pairs that were formed. I focused on the two [TEAH^+^][AcO^–^] ionic pairs whose formation takes place within the first 50 ps of the **20:20** simulation (see Figure 5). I then computed the radial distribution functions pertaining only to these two ionic pairs, excluding the frames of the trajectory previous to their formation. The results are summarized in Figure 8, where I report the O^–^ contacts (left panel) and the NH^+^ ones (right panel) with the rest of the fluid. As the relative abundances of the binding sites are the same, the height of the g(r) can be directly compared within the same panel as well as across the two panels. Most prominent is the interaction within the ionic couple, that is, the mutual interaction between the negative carboxylate and the positive ammonium group (blue line), which, obviously, turns out to be identical across the two panels. The average distance between O^–^ and N^+^ is between 2.7 and 2.8 Å, which represents a typical distance of the H-bond that binds the ionic couple. Another important feature is the fact that both the anion with O^–^ and the cation with N^+^ do not show any appreciable interaction with neutral TEA (red line). Instead, a residual, but minor interaction of the ions with neutral acetic acid (black line) can be clearly seen. In conclusion, I can say that, when the ionic pairs are formed, they are held together through electrostatic and H-bonds, and they tend to be solvated (at least at the scale of the simulations) inside the AcOH neutral moiety. I also checked that the ionic pairs remain bound (with N and O within 3.5 Å) after their formation—their computed lifetimes, obtained through a dimer autocorrelation function, simply span the entire trajectory.

### 3.5. The Other Simulations

The second simulation, **10:10**, was performed on a system of smaller size in order to explore longer timescales around 600 ps. The behavior of this system is quite similar to the one already explored in the previous sections (the **20:20** simulation). I detected only two ionization events forming [TEAH^+^][AcO^–^] ionic pairs—one occurring after 10 ps and the second one occurring at 350 ps. Different from the previous **20:20** simulation, one of two ionic pairs does not remain bound, but dissociates with O^–^ and N^+^ distances rising up to 5 Å. The initially formed AcO^–^ acquires a proton from a nearby AcOH and becomes neutral, thus breaking the ionic couple. Such an exchange of the AcO^–^ partner inside the ionic couple is an event that I did not detect in the **20:20** simulation. From the data I have, I cannot conclude if this is only a sporadic occurrence or a systematic event in the fluid.

**Figure 9:**
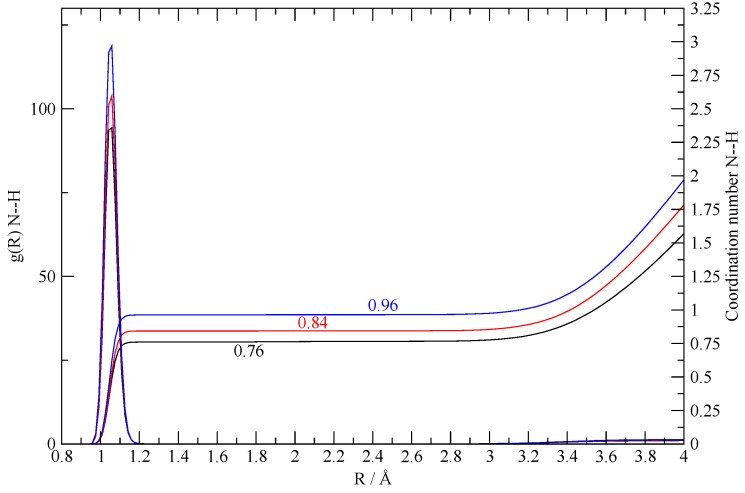
N–H g(r) in the **8:2** simulation and relative volumetric integral (that is, the coordination number) for different sampling intervals. The black lines are obtained by sampling the entire trajectory (950 ps), the red one by sampling the second half (475 ps), and the blue one by sampling the last third (320 ps).

The **8:2** simulation was prepared using an initial excess of acetic acid with a ratio of AcOH/TEA = 4:1 as in the final state of the synthesized system of [27]. The interesting fact about this simulation is that the only existing TEA molecules are quickly protonated—the first one after 50 ps and the second one after 150 ps. After this time, both TEA molecules remain bound to their AcO^–^ counterpart in an ionic couple with NH^+^–O^–^ distances around 2.5 Å. The quantitative protonation of the two TEA molecules is shown by the calculated N–H g(r) reported in Figure 9, along with the corresponding coordination number. The latter turns out to increase along the simulation, progressively reaching almost 1 when computed using only the last 320 ps of the trajectory. Such a simulation seems to indicate that, in a system with an excess of acetic acid, protonation of the TEA minor component is a complete and quantitative event.

The role of the **PI20:20** simulation is similar to that of the previous one. It can be used as a test of the stability of the system when it is prepared at the experimental degree of ionization (25% of the system is ionized). The simulation, in its 300 ps, has shown a complete absence of proton transfer, thereby providing additional evidence that, when 25% of molecules are ionized, the system remains stable.

**Figure 10:**
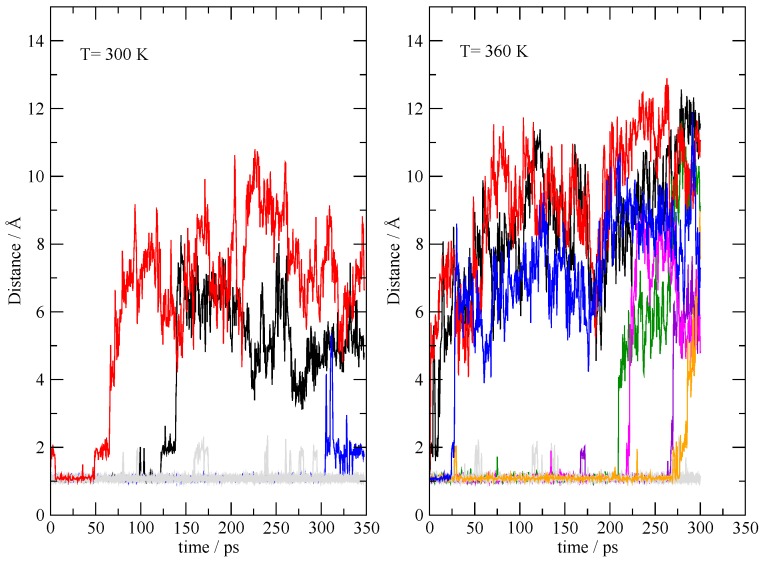
N–H distances in TEAH^+^ during the simulations initially prepared as a completely ionized system (**I20:20**). Distances were saved every 500 fs. The migrating protons are highlighted by different colors, while all the others are reported in gray.

In order to explore the (in)stability of the fully ionized state, it was simulated under the same conditions of the **20:20** simulation, starting from an initial condition where all the TEAs are protonated, and all the acetic acid are acetates. The dynamics was performed at 300 K and 360 K in order to accelerate the slow dynamics. A pictorial representation of the deprotonation process of TEAH^+^ is shown in Figure 10, where I report the 20 N–H distances within the TEAH^+^ molecules as a function of the simulation time. The migrating protons are highlighted by different colors, while all the others are reported in gray. Within the 350 ps of the simulation at 300 K, three mutual neutralization reactions can be detected where the proton migrates from the ammonium onto the acetate. The process of mutual neutralization is greatly accelerated by increasing the temperature (seven proton migrations can be seen). This means that the fully ionized state of the system is intrinsically unstable in the thermodynamic conditions sampled by the simulations.

## 4. Conclusions

In this paper, I have presented original results obtained through different bulk simulations of the liquid known as triethylammonium acetate. Despite the name that identifies it as a salt, the structure of this liquid is far from that of an ionic liquid. Recent experiments have detected only a fraction of the pure liquid actually being ionized, while the majority of it remains polar and sometimes separates into its constituents (acetic acid and triethylamine). The simulations were carried out using an MD paradigm that allows bond breaking thanks to the fact that the interatomic potential is directly obtained by a semi-empirical density functional method. Chemical topology is thus not enforced, and proton transfer processes are allowed to take place during the dynamics. Although the simulations presented here were unable to reach equilibrium owing to the relatively high computational cost, it was possible to trace a clear trend that is compatible with the experimental findings and, in particular, I have found the following: (i) starting from a completely neutral system, only a limited proton migration from the acetic acid toward the triethylamine is seen and the formation of the triethylammonium acetate ionic liquid occurs only to a limited extent; (ii) the liquid is composed by strongly aggregated neutral acetic acid, which is held together by H-bonds in which the residual ionic component is embedded; and, finally, (iii) a fully ionized mixture of acetate anions and triethylammonium cations is unstable with respect to partial mutual neutralization.
